# Next-Generation Sequencing Reveals a Novel Emaravirus in Diseased Maple Trees From a German Urban Forest

**DOI:** 10.3389/fmicb.2020.621179

**Published:** 2021-01-08

**Authors:** Artemis Rumbou, Thierry Candresse, Susanne von Bargen, Carmen Büttner

**Affiliations:** ^1^Faculty of Life Sciences, Albrecht Daniel Thaer-Institute of Agricultural and Horticultural Sciences, Humboldt-Universität zu Berlin, Berlin, Germany; ^2^UMR 1332 Biologie du Fruit et Pathologie, INRAE, University of Bordeaux, UMR BFP, Villenave-d’Ornon, France

**Keywords:** emaravirus, maple mottle-associated virus, maple virome, RNA-Seq, forest disease

## Abstract

While the focus of plant virology has been mainly on horticultural and field crops as well as fruit trees, little information is available on viruses that infect forest trees. Utilization of next-generation sequencing (NGS) methodologies has revealed a significant number of viruses in forest trees and urban parks. In the present study, the full-length genome of a novel *Emaravirus* has been identified and characterized from sycamore maple (*Acer pseudoplatanus*) – a tree species of significant importance in urban and forest areas – showing leaf mottle symptoms. RNA-Seq was performed on the Illumina HiSeq2500 system using RNA preparations from a symptomatic and a symptomless maple tree. The sequence assembly and analysis revealed the presence of six genomic RNA segments in the symptomatic sample (RNA1: 7,074 nt-long encoding the viral replicase; RNA2: 2,289 nt-long encoding the glycoprotein precursor; RNA3: 1,525 nt-long encoding the nucleocapsid protein; RNA4: 1,533 nt-long encoding the putative movement protein; RNA5: 1,825 nt-long encoding a hypothetical protein P5; RNA6: 1,179 nt-long encoding a hypothetical protein P6). Two independent NGS sequencing runs from the same symptomatic maple tree detected the same genome segments. For one of these sequencing runs the cDNA library was prepared using a primer targeting the conserved genome terminal region, known to be shared between emaraviruses genome segments. We suggest, therefore, that the six identified genome segments represent the complete genome of a novel emaravirus from maple, which we tentatively name maple mottle-associated virus (MaMaV). Phylogenetic and sequence homology analyses place this virus on the distinct “subgroup a” clade within the *Emaravirus* genus along with – among others – rose rosette virus, *Actinidia* emaravirus 2, and fig mosaic virus. Validation RT-PCR assays performed on symptomatic and non-symptomatic trees suggest that MaMaV may be the symptom-inducing virus in the diseased trees. To our knowledge, this is the first time an *Emaravirus* is described from maple and is fully genetically characterized. With the discovery of MaMaV, the genus *Emaravirus* comprising negative-sense single-stranded viruses with very divergent genomes – that were until recently overlooked – has substantially increased counting 22 established and putative members.

## Introduction

Viruses of forest trees have been, until recently, only slightly characterized due to two main reasons; (a) biased sampling based on a restricted focus on agricultural crops and fruit trees viruses (Büttner et al., 2013) and (b) a general bias against the identification of the most divergent genomes (Zhang et al., 2018). Due to the utilization of next generation sequencing (NGS), however, forest virology has gained a significant momentum in identifying viruses infecting forest trees. Recently, a birch virome was unraveled revealing a complex of novel and known viruses (Rumbou et al., 2020), while a novel badnavirus associated with the birch leaf-roll disease was identified and genetically characterized (Rumbou et al., 2018). In mosaic-diseased Eurasian aspen (*Populus tremula*) a novel emaravirus has been identified (von Bargen et al., 2020a). European mountain ash ringspot-associated virus has been detected in new hosts like *Karpatiosorbus × hybrida* in Finland (von Bargen et al., 2020b), *Sorbus intermedia* (von Bargen et al., 2019), and *Amelanchier* spp. (von Bargen et al., 2018). It is apparent, that the application of NGS tools has substantially increased the rate of virus discovery in forest and urban green ecosystems.

Viral diseases of different maple species (*Acer spp*.) have been reported by plant virologists since long (Cooper, 1979). Atanasoff (1935) was probably the first to describe a “yellow mottle-mosaic” symptom in *Acer negundo* and *A. pseudoplatanus* in Japan and Europe, possibly related to virus presence. Brierley (1944) observed chlorotic or ring mottle in *Acer saccharum* in North America, and a similar symptom was reported by Ploiaie and Macovei (1968) in Romania. Szirmai (1972) described a “mosaic mottling with chlorotic – tending to yellow ochre – spots” in *A. negundo* and *A. pseudoplatanus* in Hungary, while Cooper (1979) confirmed this symptomatology in maples in the United Kingdom. At the same time, mechanical transmission of the so-called “maple leaf perforation virus” from naturally infected maples to beans (*Phaseolus vulgaris*) was described by Subikova (1973), but without visualizing virus particles by electron microscopy (EM).

The graft-transmissibility of an agent causing leaf mottle in maples was earlier reported (Führling and Büttner, 1998), while rod-shaped virus particles approx. 300 nm-long and genomic material from tobamoviruses were observed by EM in symptomatic leaves. Rod-shaped virus particles were also detected in young *A. saccharum* seedlings with chlorotic spots and mottle symptoms (Lana et al., 1980), which were attributed to tobacco mosaic virus based on their serological and biological properties. Isometric particles of 26–30 nm diameter were detected in maple trees from Turkish urban areas (*A. negundo, A. pseudoplatanus, Acer campestre*) exhibiting mottling, mosaic, leaf deformation and lateral shoot formation (Erdiller, 1986). These particles were attributed to arabis mosaic virus, cucumber mosaic virus and soybean mosaic virus. However, to date, *Acer sp.* has never been unambiguously described as a host for any well-characterized viral agent and data of the Virus-Host database also confirm that (Mihara et al., 2016).

Maples are abundantly found in European forests and urban parks, with the most common species *A. pseudoplatanus* (sycamore) and *Acer platanoides* (Norway maple) representing a natural component of birch (*Betula* sp.) and fir (*Abies* sp.) forests (Gibbs and Chen, 2009). Several *Acer* species provide valuable timber and are the main sources of maple sugar and maple syrup (Binggeli, 1993). Damages on the trees have been regularly attributed to fungi with most harmful being *Verticillium* wilt, sooty bark disease caused by *Cryptostroma* species (Wulf and Kehr, 2009) as well as *Eutypella parasitica* causing trunk infections (Brglez et al., 2020). Phytoplasma-associated witches-broom disease has been reported in *A. negundo* (Kaminska and Suwa, 2006) as well as in Japanese maple, *Acer palmatum* (Li et al., 2012). Maple trees exhibiting virus-like symptoms were regularly observed in forests around Berlin as well as in other regions of Germany during the last 40 years (Bandte et al., 2008). However, viral agent(s) affecting maples have not been adequately characterized by conventional methods. Considering the ecological and economical importance of maples, we aimed to fill in the gap in maple’s pathology by employing an RNA-Seq methodology to identify viruses possibly affecting maple trees. As a result, the genome of a novel viral agent is fully identified and characterized, while its association with the observed symptomatology is strongly suggested.

## Materials and Methods

### Plant Materials

**FIGURE 1:**
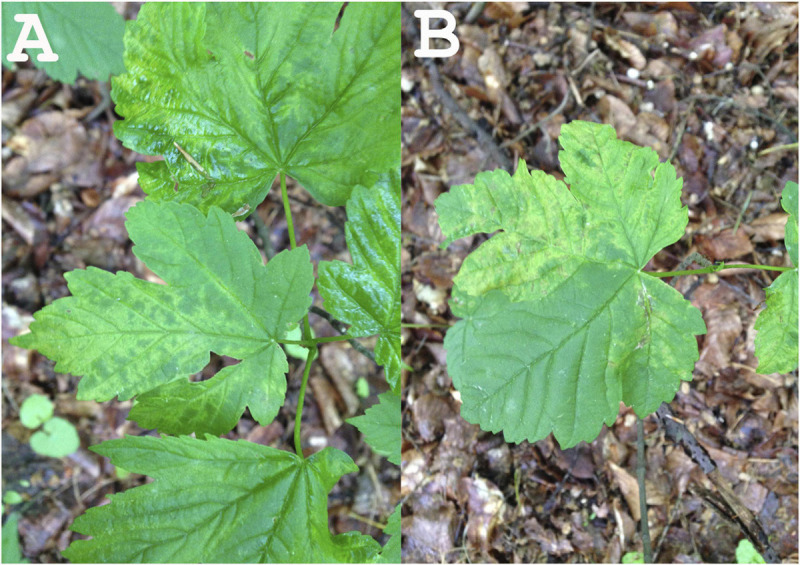
Leaf symptoms of the tested sycamore tree subjected to NGS (**A:** leaf mottle; **B:** leaf mottle and deformation).

In 2014, leaf samples exhibiting virus-like symptoms, including mottle and leaf deformation (Figure 1), were collected from an *A. pseudoplatanus* tree in the Berlin-Grunewald urban forest [Acer+ (2014): symptomatic tree], where such symptoms have been monitored for at least two decades. Randomly selected leaf parts were pooled together and used for RNA extraction. A similar pool of leaves was obtained from a symptomless seedling [Acer− (2014): non-symptomatic tree]. In 2015, the same symptomatic tree was re-sampled and RNA was extracted from pooled leaves exhibiting symptoms [Acer+ (2015)].

For the investigation of the virus presence in the urban Berlin-Grunewald forest, leaves were collected from symptomatic and non-symptomatic maples in order to be used for RNA isolation and RT-PCR diagnostic assays. In total, 26 sycamore maple trees exhibiting symptoms similar to those of the Acer+ (2014) tree as well as six trees without symptoms were tested in 2015, 2016 or 2019 (Supplementary Table 1).

### Next Generation Sequencing and Sequence Assembly

Total RNAs were isolated from 100 mg leaf tissue using the InviTrap Spin Plant RNA Mini Kit (STRATEC Molecular, Germany), followed by removal of remaining DNA with rDNase according to the supplier protocol (Macherey-Nagel, Germany) and RNA purification using NucleoSpin RNA Clean-up (Macherey-Nagel, Germany). Ribosomal RNA depletion was performed using the RiboMinus Plant Kit for RNA-Seq (Invitrogen). One to two micrograms of RiboMinus RNA of each sample were used for cDNA synthesis with the Maxima H Minus double-stranded cDNA synthesis Kit (Thermo Scientific) primed with random hexamers for samples Acer+ (2014) and Acer− (2014). For sample Acer+ (2015), the precipitated RNA was reverse transcribed into cDNA using the generic terminal primer PDAP213 (Di Bello and Tzanetakis, 2013).

Two micrograms purified double-stranded cDNA from each sample were sent to BaseClear (Netherlands) for RNA-Seq analysis on the Illumina HiSeq2500 system. FASTQ sequence files were generated using the Illumina Casava pipeline version 1.8.3. Initial quality assessment was based on data passing the Illumina Chastity filtering. Subsequently, reads containing adapters and/or PhiX control were removed using an in-house filtering protocol. The second quality assessment was based on the remaining reads using the FASTQC quality control tool version 0.10.0. NGS data processing and analysis were carried out either using CLC Genomics Workbench version 7.0.4. or the VirAnnot pipeline (Lefebvre et al., 2019). The quality of the FASTQ sequences was enhanced by trimming off low quality bases using the “Trim sequences” option of the CLC Genomics Workbench version 7.0.4. The quality-filtered reads were *de novo* assembled into contig sequences using CLC Genomics Workbench. Contigs annotation was carried out using BLASTn against the NCBI-GenBank databases. When needed, virus-related contigs were manually assembled into larger scaffolds and scaffolds polished by re-mapping of reads on the scaffolds using CLC Genomics Workbench.

### Taxonomic Analysis of the Metagenome

The taxonomic content of the obtained datasets, as provided by the BLAST annotations, was visualized using MEGAN (Huson et al., 2016), in which the BLAST results were parsed to assign the best hits to appropriate taxa in the NCBI taxonomy. As a result, the taxonomical content (“species profile”) of the sample from which the reads were collected was estimated, with a particular focus on viral species.

### Validation of the Presence of Novel Virus in Maples

Samples from 32 symptomatic and non-symptomatic maple trees were collected from two different locations in Berlin-Grunewald and from grafted scions of that origin propagated in the experimental garden of Humboldt University of Berlin since 2017 (Supplementary Table 1). Symptomatic trees exhibited most often mottle, or mottle in combination with a mixture of other symptoms like flecking, chlorotic ringspots, vein banding, chlorotic line pattern, mosaic and/or leaf deformation. Pooled samples of 100 mg leaf tissue from three to five leaves from different twigs of each tree were used. Total RNAs were isolated according to Boom et al. (1990).

To confirm the presence of the identified segments in the samples, specific RT-PCR assays were performed using segment-specific primer pairs (Table 1). The first-strand cDNAs were synthesized from 1 μg of total RNA in a 20 μl reaction volume of 1x RT buffer (Thermo Scientific) containing 1 μM dNTPs mix, 100 U RevertAid Premium reverse transcriptase (Thermo Scientific), 20 U RiboLock RNase inhibitor (Thermo Scientific), and 100 pmol of random hexamer-oligonucleotides (Biolegio). Subsequent PCR amplifications were conducted in a 50 μl volume of 1x DreamTaq Buffer (Thermo Scientific) containing 0.2 μM dNTP mix, 0.25 U of DreamTaq DNA polymerase and 1 μM of each forward and reverse primer. The thermal cycles were as follows: 2 min at 94°C followed by 35 cycles at 94°C for 30 s, 55°C for 30 s, 72°C for 30 s, with a final extension step of 72°C for 5 min. The product lengths amplified for the different RNA segments are shown in Table 1.

**TABLE 1:** Features of primers used for the specific RT-PCR detection of MaMaV.

**Primer name**	**Primer sequence (5**′ **– 3**′)	**Product length (bp)**
**RNA1aF**	AACCAATGCTGTCACTTAAGC	346
**RNA1aR**	GATATAACTACCATCTAACATCC	
**RNA1bF**	GGATGTTAGATGGTAGTTATATC	290
**RNA1bR**	CCACTTATAGTTATTGCTTCACC	
**RNA2F**	GCAAGATTTTGATGTGGCTGG	149
**RNA2R**	AACCATCATGGCCATCACAAC	
**RNA3F**	TGTGCTATAATGGCAGCTGG	289
**RNA3R**	CATCAGTCATGCTATCTGGTATG	
**RNA4F**	TTGGACACCAACATCTACAAG	470
**RNA4R**	GCAATTCCTTCCTCTCATTGT	
**RNA5F**	GAACTATGTCTTACCAACACTG	221
**RNA5R**	CTAATTCCCTAAGTTTGATAGTAAC	
**RNA6F**	CAGATAACATATTCTCTTCTGG	300
**RNA6R**	AAGCGAGATATATGCTATGGCT	

### Sequence Analysis and Phylogenetic Comparison of Novel Sequences

Multiple nucleotide or amino acid sequence alignments as well as pairwise sequence identity calculations were performed using AliView version 1.17.1 (Larsson, 2015). ORF finder at NCBI^1^ was used to identify open reading frames (ORFs) on assembled genome segments and identify the encoded proteins. All ORFs with 300 or more nucleotides (nt) were considered. For the phylogenetic comparisons of complete coding regions, the 21 established and tentative emaravirus species identified to date and represented in GenBank were used. Maximum likelihood (ML) trees were constructed with MEGA6 (Tamura et al., 2013) applying the Jones-Taylor-Thornton (JTT) substitution model for amino acids. Robustness of nodes of the phylogenetic tree was assessed from 1,000 bootstrap replications and values >70% were displayed for trees’ internal nodes.

To perform preliminary genetic divergence analysis, 40 RT-PCR products from 10 tested samples were directly submitted for Sanger sequencing (Macrogen) without previous cloning. They were amplified from three RNA segments; RNA1, RNA3, and RNA4. For RNA1, RT-PCR products were amplified in two different genome regions; the primer-pair RNA1aF/R generated PCR products of 311 nt length (nt positions 3,006 – 2,756), while the primer pair RNA1bF/R generated 274 nt-long RT-PCR products located at nt positions 3,317 – 3,042. Sequences of RT-PCR products for the RNA3 segment were 274 nt-long (nt positions 734 – 468), while in the RNA4 segment RT-PCR products of 431 nt length were amplified (nt positions 835 – 405). Evolutionary analyses were conducted in MEGA6 (Tamura et al., 2013). Genetic distance between sequences was assessed applying the Maximum Composite Likelihood model (Tamura et al., 2004), where the number of base substitutions per site between sequences was calculated.

## Results

### Quality Analysis of FASTQ Sequence Reads, *de novo* Assembly and Taxonomic Analysis of the Metagenome

RNA-Seq was performed in 2014 using RNA preparations from a symptomatic and a symptomless maple tree. 124 and 14 MB data/sample with average quality of approx. 35 Phred were generated for Acer+ (2014) and Acer− (2014), respectively [620,460 FASTQ reads for Acer+ (2014); 69,353 FASTQ reads for Acer− (2014)] (Table 2). For the sample Acer+ (2014) the *de novo* assembly of quality-filtered paired-end reads resulted in 532 assembled contigs. Analysis identified 14 contigs exhibiting significant nt identities to RNA sequences of several emaraviruses as assessed by BLASTn (seven contigs with identities to emaravirus-RNA1 assembling a 6,005 bp-long scaffold with missing genome parts within the sequence and at the ends; three contigs with identities to emaravirus-RNA2 assembling a 1,761 bp-long scaffold; one contig 1,085 bp-long with identities to emaravirus-RNA3; one 1,256 bp-long contig with identities to emaravirus-RNA4; one 1,277 bp-long contig with identities to RNA5 segment of some emaravirus species; one 928 bp-long contig with identities to RNA6 segment of some emaravirus species). All assembled contigs/scaffolds were missing sequences at the 3′ and 5′ ends. In the negative control sample Acer− (2014) none of the 30 contigs assembled showed any significant identities to emaraviruses or any other plant viruses. No other contigs from the Acer+ (2014) sample were identified as viral besides the 14 ones showing affinities with emaraviruses.

**TABLE 2:** Quality statistics of FASTQ sequence reads and *de novo* statistics for the three maple samples.

**Plant**	**FastQ sequence reads**	**Sample Yield (in MB)**	***De novo* assembly**				
			**Total reads**	**Matched reads**	**Total contigs**	**Average contig length (bp)**	**Emara-specific contigs**
**Acer+ (2014)**	620,460	124	1,116,554	924,998	532	677	14
**Acer− (2014)**	69,353	14	123,424	115,795	30	984	0
**Acer+ (2015)**	850,283	198	1,700,566	1,496,745	2,206	646	8

The contigs resulting from the *de novo* assembly of the reads for each sample were used for the MEGAN analysis. For the symptomatic sample Acer+ (2014), out of the 532 contigs assembled, 474 belong to Eucaryota, most of them to clade *Euphylophyta* (Phylum: Streptophyta; Kingdom: Viridiplantae) – where *Acer spp*. is classified – and three belong to Bacteria. The 11 viral contigs identified by MEGAN are attributed to the *Fimoviridae* family and ten are attributed to pigeon pea sterility mosaic virus (*Emaravirus*, *Fimoviridae*). In the case of the non-symptomatic sample Acer− (2014), none of the 30 assembled contigs is attributed to a viral species. The taxonomic analysis performed by MEGAN show a high degree of consistency with the results delivered by BLASTn annotation and clearly suggest the presence of an emaravirus in the tested samples.

**FIGURE 2:**
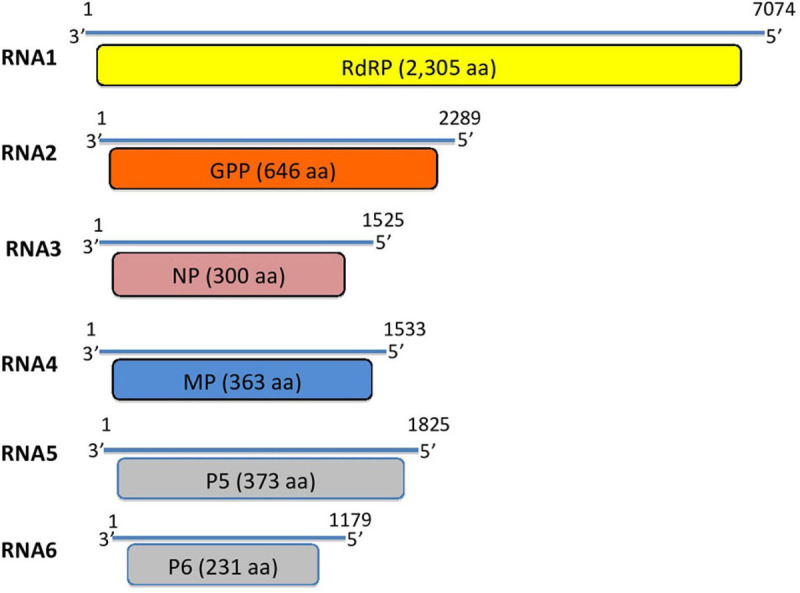
Genome segments of the novel maple mottle-associated virus (MaMaV). RdRP, RNA-dependent RNA polymerase; GPP, glycoprotein precursor; NP, nucleocapsid protein; MP, movement protein; P5, hypothetical protein; P6, hypothetical protein. Numbers represent the nucleotide positions of the RNA segments.

To confirm the RNA-Seq results from the Acer+ (2014) sample and to complete the missing parts of the detected genome segments, the whole NGS process was repeated in 2015 with leaves from the same tree. A ds-cDNA library was generated using the emara-specific terminal primer PDAP213, aiming to detect the missing genome ends. The RNA-Seq analysis provided a higher amount of sequence data (198 MB), delivering 850,283 FASTQ sequence reads (av. quality score: ∼35 Phred) (Table 2). The *de novo* assembly resulted in a total of 2,206 contigs with an average length of 646 bp. BLASTn analysis identified eight long contigs exhibiting significant identities to emaraviruses (three with identities to emaravirus-RNA1, assembling a 6,907 bp-long scaffold missing the 5′ end; one 2,289 bp-long contig with identities to emaravirus-RNA2; one 965 bp-long contig with identities to emaravirus-RNA3; one short 235 bp-long contig with identities to emaravirus-RNA4, one 1,851 bp-long contig with identities to RNA5 segment of some emaraviruses, and one 1,100 bp-long contig with identities to RNA6 segment of some emaravirus). The new contigs showed 100% nucleotide identity with the ones generated from the same tree in 2014. As such, they were assembled together with the 2014 contigs, providing the full-length sequence of the various RNA segments. Finally, a polishing step was performed to ensure genome completion and the absence of errors and to confirm genome ends. As a result, complete sequence of six genomic RNA segments were obtained (RNA1: 7,074 nt; RNA2: 2,289 nt; RNA3: 1,525 nt; RNA4: 1,533 nt, RNA5: 1,825 nt; RNA6: 1,179 nt) (Figure 2). The full-length genomic sequences of the maple emaravirus RNA segments are deposited in GenBank under accession numbers MT879190–MT879195.

### Genome Structure and Encoded Proteins

To identify ORFs on assembled genome segments, ORF Finder from NCBI was employed and identified six ORFs, one corresponding to each RNA segment. BLASTp annotation of the amino acid (aa) sequences derived from the assembled scaffolds from the symptomatic maple tree revealed high BLAST scores with RNA segments from viruses of the genus *Emaravirus* (*Fimoviridae, Bunyavirales*) (Table 3).

**TABLE 3:** Pairwise comparison of sequence identities at the amino acid level of putative proteins (RdRP, GPP, NP, MP, P5, P6) encoded by RNA1-RNA6 of the novel *Emaravirus* from *Acer pseudoplatanus* with the homolog proteins of the established and putative members of the genus *Emaravirus*.

		**RdRP**	**GPP**	**NP**	**MP**	**P5**	**P6**
1	Rose rosette virus	**74.40%**	**56.65%**	51.77%	64.84%	**44.81%**	42.04%
		QHZ99251.1	QID76023.1	QIB97971.1	QIB98058.1	QIB98219.1	QJR96770.1
2	Aspen mosaic-associated virus	70.23%	53.11%	51.04%	57.73%	–	40.39%
		CAA0079389.1	CAA0079597.1	CAA0079646.1	CAA0079685.1		CAA0079719.1
3	*Actinidia* emaravirus 2	69.66%	47.78%	**55.63%**	63.31%	43.41%	**44.16%**
		QEE82886.1	QEE82887.1	QEE82888.1	QEE82889.1	QEE82890.1	QEE82891.1
4	Pistacia emaravirus	69.16%	52.07%	51.60%	62.33%	40.46%	32.43%
		QAR18002.1	QAR18003.1	QAR18004.1	QAR18005.1	QAR18006.1	QAR18008.1
5	Fig mosaic emaravirus	68.67%	52.15%	54.04%	**65.19%**	33.98%	41.71%
		QBH72675.1	QBK46595.1	BAM13809.1	BAM13817.1	YP_009237273.1	BAM13854.1
6	Pigeon pea sterility mosaic emaravirus 1	53.58%	46.56%	40.29%	62.64%	42.92%	37.70%
		ANQ90714.1	QBA83603.1	CDX09880.1	ANQ90777.1	CUR49054.1	ANQ90719.1
7	Pigeon pea sterility mosaic emaravirus 2	68.14%	51.32%	53.29%	64.00%	43.89%	37.70%
		QBA83607.1	YP_009268865.1	ALU34071.1	ANQ90759.1	QBA83611.1	ANQ90763.1
8	Blackberry leaf mottle-associated virus	66.09%	52.22%	50.17%	56.32%	–	35.57%
		AQX45473.1	AQX45474.1	AQX45475.1	QBM15152.1		AQX45477.1
9	European mountain ash ringspot-associated emaravirus	48.70%	40.67%	36.69%	35.24%	–	–
		VFU05375.1	YP_003104765.1	SPN63240.1	VFU05382.1		
10	Actinidia chlorotic ringspot-associated virus	47.82%	41.53%	38.43%	33.70%	–	–
		YP_009507925.1	YP_009507926.1	YP_009507928.1	YP_009507927.1		
11	Lilac chlorotic ringspot-associated virus	48.92% QIN85945.1	42.61%	40.69%	36.56%	–	–
			QIN85946.1	QIN85947.1	QIN85948.1		
12	Redbud yellow ringspot-associated emaravirus	47.27%	40.96%	37.91%	35.85%	–	–
		YP_009508083.1	YP_009508087.1	YP_009508085.1	YP_009508084.1		
13	Ti ringspot-associated emaravirus	37.26%	26.55%	30.82%	23.10%	–	–
		QAB47307.1	QAB47308.1	QAB47309.1	QAB47310.1		
14	Raspberry leaf blotch emaravirus	36.87%	26.98%	28.57%	24.08%	31.91%	–
		YP_009237274.1	YP_009237265.1	YP_009237266.1	YP_009237267.1	YP_009237268.1	
15	Palo verde broom virus	35.24%	26.42%	24.42%	27.00%	–	–
		AWH90165.1	AWH90170.1	AWH90176.1	AWH90182.1		
16	Jujube yellow mottle-associated virus	37.60%	28.39%	28.23%	24.53%	–	–
		QDM38999.1	QDM39000.1	QDM39001.1	QDM39002.1		
17	High Plains wheat mosaic emaravirus	34.97%	28.17%	26.55%	24.14%	33.33%	–
		YP_009237277.1	QGT41075.1	YP_009237257.1	QGT41077.1	QGT41070.1	
18	Camellia japonica-associated emaravirus 1	32.61%	26.60%	29.73%	19.93%	–	–
		QGX73503.1	QGX73504.1	QGX73505.1	QGX73506.1		
19	Camellia japonica-associated emaravirus 2	32.89%	25.93%	25.85%	21.07%	–	–
		QGX73507.1	QGX73508.1	QGX73509.1	QGX73510.1		
20	Perilla mosaic virus	32.07%	23.75%	32.58%	21.36%	–	*–*
		BBM96177.1	BBM96178.1	BBM96180.1	BBM96181.1		
21	Common oak ringspot-associated emaravirus	35.73%	28.04%	27.27%	–	–	*–*
		LR828198	LR828199	LR828200			
22	Pea-associated emaravirus	–	–	50.52%	–	–	–
				QJX15716.1			

**FIGURE 3:**
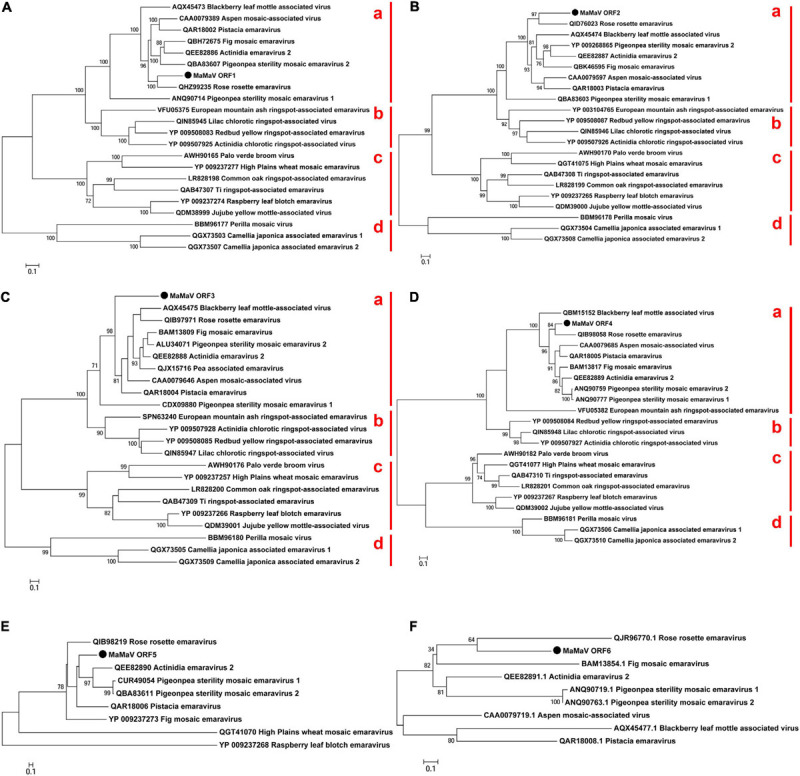
Phylogenetic tree reconstructed using the amino acid sequences of the RdRP **(A)**, GPP **(B)**, NP **(C)**, MP **(D)**, P5 **(E)**, and P6 **(F)** of the MaMaV. The analysis involved 22 amino acid sequences of established and putative members of the genus *Emaravirus* (the GenBank accession numbers are also indicated). The tree was constructed using the Maximum Likelihood method and the statistical significance of branches was evaluated by bootstrap analysis (1,000 replicates). Only bootstrap values above 70% are indicated. The scale bar represents 10% amino acid divergence. Subgroups a and b described by Elbeaino et al. (2018) as well as subgroups c and d within the genus *Emaravirus* are indicated at the right side of the trees.

ORF1 predicted on RNA1 is 2,305 aa-long and the encoded protein shows in BLASTp analysis significant aa identity of 32–75% with the viral replicase of 21 known emaraviruses (ORF1: nt positions 6,966 – 49). The putative RNA polymerase exhibits highest aa identity with that of rose rosette virus (Accession number: QHZ99251.1; 74.4% aa identity) (Figure 3).

ORF2 encoded on RNA2 is predicted to encode a protein of 646 aa (nt positions 1,996 – 56). In BLASTp comparisons the encoded protein shows 26–57% aa identity with 21 other emaraviruses. The highest aa identity is with the glycoprotein precursor (GPP) of rose rosette virus (Accession number: QID76023.1; aa identity: 56.7%).

ORF3 encoded on RNA3 is predicted to encode a protein of 300 aa (nt positions 104 – 1,006) with highest BLASTp aa identity with the nucleocapsid proteins (NP) of the 21 known emaraviruses (24–56%). The ORF3-encoded protein shows highest aa identity with the NP protein of *Actinidia* emaravirus 2 (Accession number: QEE82888.1; aa identity: 55.6%). In Table 3 the recently identified NP protein from pea-associated virus (Y.Z.A Gaafar and Ziebell, 2019, H; GenBank: QJX15716.1.) is also included, because ORF3 shows relatively high identity with it (50.5%). There are, however, no other proteins identified from this tentative emaravirus.

ORF4 encoded on RNA4 is predicted to encode a protein of 363 aa (nt positions 1,179 – 88) which shows very variable BLASTp aa identity levels with the movement proteins identified from 20 emaraviruses (19–65%). It exhibits highest aa sequence identity with the movement protein of fig mosaic virus (Accession number: BAM13817.1; aa identity: 65.2%).

The fifth predicted ORF is encoded on RNA5 and is predicted to encode a protein of 373 aa (nt positions 1,526 – 93). The hypothetical protein encoded by RNA5 shows sequence homology (supported by *E*-values < 0.01) with the corresponding protein detected from eight emaraviruses (30–43% aa identity). This putative protein shares highest identity with the P5 protein of rose rosette virus (Accession number: QIB98219. 1; aa identity: 44.8%), a protein of unknown function. The rest of the emaraviruses do not have a homologous protein or such has not been identified yet.

Finally, the sixth ORF, encoded on RNA6 (nt positions 766 – 71), produces a 231 aa-long protein with 35–45% aa identity with that of other emaraviruses. The highest aa identity is with the P6 protein from *Actinidia* emaravirus 2 (Accession number: QEE82891.1; aa identity: 44.2%). The novel virus is found to have a homologous protein to the one encoded by RNA6 segment of only eight from the already known emaraviruses.

Elbeaino et al. (2018) in the 10th report of the International Committee on Taxonomy of Viruses regarding the taxonomy of *Fimoviridae* describe that the virus genome of the family members comprises from four negative-sense ssRNA segments [like the type species European mountain ash ringspot-associated emaravirus (EMARaV) from the host European mountain ash] to eight RNA segments [High Plains wheat mosaic virus (Tatineni et al., 2014)]. Later, two novel emaravirus with five RNA segments were identified; lilac chlorotic ringspot-associated virus from lilac leaves with yellow mottle symptoms in China (Wang et al., 2020) and ti ringspot-associated virus from the ti plant (*Cordyline fruticosa*) in Hawaii (Olmedo-Velarde et al., 2019). Six genomic RNA segments were identified in jujube yellow mottle-associated virus causing the same named disease in jujube (*Ziziphus jujuba*) in China (Yang et al., 2019). Very recently, a novel emaravirus in Shiso named *Perilla mosaic virus* (PerMV) was found to consist of 10 RNA segments, each encoding a single protein in the negative-sense orientation (Kubota et al., 2020). In the case of the maple virus two independent NGS samples from the same maple tree detected the same number of genome segments. Additionally, for the Acer+ (2015) sample, the cDNA library was prepared using a primer targeting the conserved genome terminal region, known to be shared among emaraviruses genome segments. A screen for further genome segments containing these terminal regions did not reveal further sequences and we therefore consider that it is unlikely to have missed additional RNA segments of the viral genome. Concluding, we suggest that the segments RNA1-RNA6 represent the complete genome of the novel virus constituting a new member of the family *Fimoviridae*.

### Phylogenetic Analysis of the Novel Emaravirus

Phylogenetic relationships between the maple virus and the sequences of 21 emaraviruses known to date were estimated, based on amino acid sequences comparisons. Irrespective of the RNA segment investigated, the maple viral agent clusters consistently in “subgroup a” (according to Elbeaino et al., 2018), together with – among others – rose rosette virus, fig mosaic virus, the novel aspen mosaic-associated virus, the two pigeon pea sterility mosaic emaraviruses and *Actinidia* emaravirus 2. The phylogenetic analysis performed here confirms the overall structure within the genus *Emaravirus* described by von Bargen et al. (2020a) and enriches the tree with seven additional emaraviruses. Figures 3A–D shows representative ML trees obtained using the putative RdRP, the GPP, the NP and the MP encoded by the genome segments RNA1, RNA2, RNA3, and RNA4, respectively. The maple emaravirus proteins P5 and P6 are homologous to those of eight different emaraviruses (Figures 3E,F). All predicted proteins cluster together with the respective ones from rose rosette virus. The only exception is the NP protein, which still clusters with the “subgroup a,” but with no close relation to any of its members. Additionally, a fourth subgroup is constructed (“subgroup d”) based on the phylogenetic relationships inferred from ORF1 – ORF4 amino acid sequences, which consists of three recently discovered viruses, the perilla mosaic virus and the camellia japonica-associated emaraviruses 1 and 2.

The novel aa sequences, as it is shown from the phylogenetic analysis, are clearly genetically differentiated from all encoded proteins identified to date and cluster together with the ones from the “subgroup a.” This result was already drawn by the taxonomic analysis with MEGAN, where the novel virus was shown to be close related to the “subgroup a” member pigeon pea sterility mosaic emaravirus (PPSMV) (without differentiation between PPSMV-1 and PPSMV-2). At the same time, all predicted proteins of the maple emaravirus exhibit significant aa identities only with the ones from other emaraviruses. Taken together, these results demonstrate that the virus identified represents a new species in the genus *Emaravirus* and it is, therefore, tentatively named maple mottle-associated virus (MaMaV).

### Validation of the RNA-Seq Results and Association of Virus Presence With Symptom Appearance

From the 32 maple trees tested by specific RT-PCR, MaMaV was only detected in the symptomatic ones and all six RNAs were generally simultaneously detected (Supplementary Table 1). For a few samples, one of the six RNA segments failed to be amplified [i.e., RNA2 in sample E54934; RNA1 (using primers RNA1bF/R) in samples E54936 and E54942 (although primers RNA1aF/R did produce amplicons with these samples); RNA5 in sample E54946]. Because these cases were rare and all other RNA segments in those samples were successfully amplified, we suggest the negative results were due to the low concentration of respective RNA segments beyond detection limit of the RT-PCR assay. Non-symptomatic samples were consistently negative for all MaMaV segments. These results suggest that the 26 positive-tested symptomatic maples were MaMaV-infected and that MaMaV could be the virus responsible for the identified virus-like symptoms.

It should be, however, underlined that the tested trees showed a variability of symptoms, where mottle was mainly observed, but other -weaker or stronger- symptoms were also exhibited. Although mottle was the main and common symptom exhibited in the majority of infected trees, occasionally other virus-like symptoms, like chlorotic ringspots, line pattern or flecking and in some rare cases also leaf deformations were observed. More concretely, apart from mottle, in 2015 also flecking was recorded, while in 2016 chlorotic ringspots and line pattern were observed, although in both years samples were collected at the same time (1st of July; see Supplementary Table 1).

### Preliminary Results on Genetic Variability of Three RNA Segments

The evolutionary divergence between variants from different trees was estimated for three RNA segments. In these RNA segments low genetic diversity was estimated; maximal divergence among RT-PCR products amplified with primer-pairs RNA1aF/R and RNA1bF/R was 2% and 1.1%, respectively. Sequences were identical in two and in 13 of the pairwise comparisons for the RNA1aF/R and RNA1bF/R RT-PCR products, respectively. However, all sequences differed from the corresponding variant generated from the original sequence from Acer + (2014) by 0.3–1.6% and by 0.4–1.1% in the cases of the RT-PCR products amplified by primer-pair RNA1aF/R and RNA1bF/R, respectively (Supplementary Table 2). Regarding variability among RT-PCR products for the RNA3 and among RT-PCR products from the RNA4 segment, no genetic diversity was shown among the respective sequences and all were identical with the corresponding segment from the original sample Acer+ (2014).

Based on these results, evidence is provided that the diversity in the local Berlin-Grunewald MaMaV population is generally low and that different level of genetic diversity may characterize its different genome segments.

## Discussion

The six newly identified RNA segments are attributed to a novel *Emaravirus* species based on the following: (a) The multipartite genome is composed of six single-stranded RNA molecules; (b) All six RNAs share a fully conserved stretch of 13 nt at their 5′ and 3′ termini; (c) Each segment of the genome encodes a single protein, which shows sequence identity with homologous proteins of other emaraviruses; (d) In all phylogenetic trees generated with amino acid sequences, MaMaV is only distantly related phylogenetically to the emaraviruses currently represented in the GenBank fulfilling the current species demarcation criteria of emaraviruses to show more than 25% aa divergence of RNA1-RNA3 encoded proteins (Elbeaino et al., 2018). To our knowledge, for first time an emaravirus is described from maple and is fully genetically characterized.

The NGS method applied not only led to the discovery and genetic characterization of the novel emaravirus; it unraveled at the same time the maple virome, in terms of identifying the exhaustive collection of nucleic acid sequences deriving from viral agents. The maple virome of the symptomatic tree tested is found to be very simple, as it includes a single variant from a single virus. The lack of virome complexity is rather surprising, when we consider obtained NGS results from other wild as well as cultivated woody hosts. A complex virome was revealed in birch, where up to five virus variants were identified in the transcriptome of individual trees (Rumbou et al., 2020). In birch, it has been demonstrated that not only multiple viral species but also diverse variants of the same virus may accumulate in single trees (Rumbou et al., 2016, 2020). Similarly, in single peach trees multi-viral infection of up to six viruses and viroids was detected (Jo et al., 2018). A multiplicity of viral infections was also shown in metagenome samples from single plum trees (Jo et al., 2020). A possible explanation for the single viral infection in the maple tree could be the age of the tree; it was very young (approx. 3 years-old) when sampled, thus it was exposed for only a short time to viral pathogens.

The novel virus was only present in the tested symptomatic maple trees in Berlin, while it was not detected in non-symptomatic trees. This suggests that MaMaV presence is associated with the leaf symptoms identified in maples. Maple seedlings scions from Berlin-Grunewald were grafted to non-symptomatic 2-year old maple rootstocks in March 2017 (data not shown). In 2018 the grafted seedlings exhibited mottle, while MaMaV could be detected in the scions by RT-PCR in 2019 and in 2020. Detection of MaMaV in rootstocks was not yet possible, as either the grafted seedlings did not develop shoots from rootstocks, or non-symptomatic shoots from rootstocks were not tested. Although the main observed symptom was mottle, other -weaker or stronger- symptoms were also exhibited. Whether MaMaV is the only causal agent for the different kind of symptoms or whether other viruses are also involved in the symptomatology needs to be further investigated. Further efforts are needed to satisfy Koch’s postulates and firmly establish its causal role.

In maple trees, virus-like symptoms other than mottle have been observed and reported earlier not only in Germany (Bandte et al., 2008; Büttner et al., 2013), but also in Romania (Ploiaie and Macovei, 1968), Hungary (Szirmai, 1972), Turkey (Erdiller, 1986), North America (Brierley, 1944), United Kingdom (Cooper, 1979). Symptom descriptions such as “mosaic,” “chlorotic mottle,” “yellow-mottle,” “ring-mottle,” “mosaic mottling with chlorotic spots” were used by earlier researchers. Whether the newly identified virus might be associated with the symptoms described in these earlier studies remains to be determined, given in particular that electron-dense structures known as double-membrane-bound bodies with diameters differing within the range of 80–200 nm and typical of emaraviruses (Mielke-Ehret and Mühlbach, 2012) have never been reported in earlier electron microscopy samples from symptomatic maples, while more typical plant virus particles sometimes have. Furthermore, infection of maples by tobamoviruses reported by Führling and Büttner (1998) could not be confirmed by the present study. In that case, we suggest either that the samples observed by EM were contaminated during sample processing or that indeed tobamoviruses may also affect maples. It remains to be confirmed by future NGS analyses whether other viral agents may be present in symptomatic maples.

From the discovery of the first viral agent in maple thanks to the development of NGS’s tools to its full biological characterization there is a long way to go (Massart et al., 2017). To investigate its potential for emergence, possible vectors and mode(s) of dispersal need to be determined. Several members of the genus *Emaravirus* are known to be transmitted by eriophyid mites (*Acar*i: *Eriophyidae*) (Mielke-Ehret and Mühlbach, 2012; Hassan et al., 2017; Elbeaino et al., 2018). In the present study, in several of the sampled trees damages from gall mites (*Aceria macrophylla*, *Eriophyes psilomerus*) as well as from leafhoppers were found. The gall mites can be considered as putative vectors, but the hypothesis that they may be involved in the emaravirus transmission needs to be studied. Regarding MaMaV’s impact on infected trees, we assume that the mottle symptoms exhibited in infected leaves may lead to reduced photosynthetic capacity and, consequently, to trees’ health deterioration. Based on the available diagnostic RT-PCR assays designed in the frame of the present study, the investigation of the agents’ distribution and its impact on the trees health can be estimated.

During the last decade a significant number of novel DNA and RNA viruses (Villamor et al., 2019) and viroids (Hadidi, 2019) have been uncovered in herbaceous as well as in woody hosts by applying a wide range of NGS methods for virus detection and discovery (Hadidi et al., 2016; Olmos et al., 2018; Villamor et al., 2019). In comparison to other viruses, emaraviruses were until recently overlooked, not only when applying conventional methods, but also by metagenomic studies (Bejerman et al., 2020). The application of NGS resulted in the discovery of 13 emaraviruses during the last 5 years (Bejerman et al., 2020). By employing NGS methodologies a significant presence of emaraviruses in forests has been revealed. In the last 3 years four emaraviruses have been discovered in six forest or urban tree species; aspen mosaic-associated virus (AsMaV) in *Populus tremula* (von Bargen et al., 2020a), European mountain ash ringspot-associated virus (EMARaV) in *Sorbus intermedia* (von Bargen et al., 2019), *Karpatiosorbus × hybrid* (von Bargen et al., 2020b), and *Amelanchier* sp. (von Bargen et al., 2018), common oak ringspot-associated virus (CORaV) in *Quercus robur* (Bandte et al., 2020), and maple mottle-associated virus (MaMaV) in *Acer pseudoplatanus*. Based on existing knowledge on viral disease of fruit trees (Hadidi et al., 2011) we suggest that viral agents might be a considerable stress factor for forests trees, they may predispose affected trees to other more harmful stress factors and, as a consequence, lead to deterioration of forests and other natural ecosystems (Büttner et al., 2013, 2015). Additional efforts in the field of forest virology are needed to provide data on the magnitude of the tree damage due to viral infections and to prevent future viral outbreaks.

## Data Availability Statement

The datasets presented in this study can be found in online repositories. The names of the repository/repositories and accession number(s) can be found below: https://www.ncbi.nlm.nih.gov/, MT879190–MT879195.

## Author Contributions

AR and CB: conceptualization. AR and TC: data curation, formal analysis, and software. CB: funding acquisition and project administration. AR, TC, and SB: investigation and validation. AR and SB: methodology and visualization. AR: writing – original draft. TC, CB, and SB: Writing – review and editing. All authors contributed to the article and approved the submitted version.

## Conflict of Interest

The authors declare that the research was conducted in the absence of any commercial or financial relationships that could be construed as a potential conflict of interest.

## Footnotes

1 https://www.ncbi.nlm.nih.gov/orffinder/

## Supplementary Material

Supplementary Table 1 Samples used for RT-PCR validation and genetic divergence analysis. Sample label, collection date, tree location, symptoms exhibited, damage caused by pests and the RT-PCR results (+/−) for each of the RT-PCR assays applied with seven different primer-pairs in 6 RNA segments of the novel emaravirus are shown.

Supplementary Table 2 Estimates of evolutionary divergence among RT-PCR products from 10 maple trees and the original Acer+ (2014). **(A)** RT-PCR products amplified with primer-pair RNA1aF/R. **(B)** RT-PCR products amplified with primer-pair RNAbF/R.
