# Supplementary material for: Next-Generation Sequencing Reveals a Novel Emaravirus in Diseased Maple Trees From a German Urban Forest
Source: Front Microbiol. 2021 Jan 8;11:621179. doi: 10.3389/fmicb.2020.621179 (PMC7819872; doi:10.3389/fmicb.2020.621179)
Supplement: Supplementary Table 1 — Samples used for RT-PCR validation and genetic divergence analysis. Sample label, collection date, tree location, symptoms exhibited, damage caused by pests and the RT-PCR results (+/−) for each of the RT-PCR assays applied with seven different primer-pairs in 6 RNA segments of the novel emaravirus are shown. [file Table_1.DOC]

**Supplementary Table S1. Samples used for RT-PCR validation and genetic divergence analysis. Sample label, collection date, tree location, symptoms exhibited, damage caused by pests and the RT-PCR results for each of the RT-PCR assays applied with seven different primer-pairs in 6 RNA segments of MaMaV are shown.**

| **Sample** | **Collection date** | **Location** | **Symptom(s)** | **Damage caused by pests** | **MaMaV-RNA1aF/R** | **MaMaV-RNA1bF/R** | **MaMaV-RNA2** | **MaMaV-RNA3** | **MaMaV-RNA4** | **MaMaV-RNA5** | **MaMaV-RNA6** |
| --- | --- | --- | --- | --- | --- | --- | --- | --- | --- | --- | --- |
| E54415 | 01/07/2015 | Grunewald | mottle, flecking, few chlorotic ringspots | *Aceria macrorhyncha*, sucking damage from leaf hopper | + | + | + | + | + | + | + |
| E54416 | 01/07/2015 | Grunewald | mottle, flecking | sucking damage from leaf hopper | + | + | + | + | + | + | + |
| E54417 | 01/07/2015 | Grunewald | mottle |  | + | + | + | + | + | + | + |
| E54418 | 01/07/2015 | Grunewald | mottle |  | + | + | + | + | + | + | + |
| E54419 | 01/07/2015 | Grunewald | mottle, weak flecking |  | + | + | + | + | + | + | + |
| E54420 | 01/07/2015 | Grunewald | mottle | *Aceria macrorhyncha* | + | + | + | + | + | + | + |
| E54423 | 01/07/2015 | Grunewald | mottle |  | + | + | + | + | + | + | + |
| E54424 | 01/07/2015 | Grunewald | mottle, some areas with vein banding |  | + | + | + | + | + | + | + |
| E54442 | 01/07/2015 | Grunewald | - |  | nt | - | - | - | - | - | - |
| E54934 | 01/07/2016 | Grunewald | mottle, chlorotic ringspots | *Aceria macrorhyncha* | +  RJ312** | +  RJ322** | - | +  RJ332** | +  RJ342** | + | + |
| E54935 | 01/07/2016 | Grunewald | mottle, some areas with chlorotic ringspots |  | +  RJ313** | +  RJ323** | + | +  RJ333** | +  RJ343** | + | + |
| E54936 | 01/07/2016 | Grunewald | mottle, clorotic line pattern | *Aceria macrorhyncha* | nt | - | + | + | + | + | + |
| E54937 | 01/07/2016 | Grunewald | mottle, clorot. ringspots, some parts with line pattern | Aceria macrorhyncha, sucking damage from leaf hopper | +  RJ314** | +  RJ324** | + | +  RJ334** | +  RJ344** | + | + |
| E54938 | 01/07/2016 | Grunewald | mottle |  | +  RJ315** | +  RJ325** | + | +  RJ335** | +  RJ345** | + | + |
| E54939 | 01/07/2016 | Grunewald | mottle | Aceria macrorhyncha, sucking damage from leaf hopper | +  RJ316** | +  RJ326** | + | +  RJ336** | +  RJ346** | + | + |
| E54940 | 01/07/2016 | Grunewald | strong mottle, chlorot. ringspots, chlorotic line pattern | *Aceria macrorhyncha* | nt | + | + | + | + | + | + |
| E54941 | 01/07/2016 | Grunewald | mottle, chlorotic ringspots | *Aceria macrorhyncha* | nt | + | + | + | - | + | + |
| E54942 | 01/07/2016 | Grunewald | chlorotic ringspots |  | nt | - | + | + | + | + | + |
| E54943 | 01/07/2016 | Grunewald | mottle, chlorot. ringspots, beginning of leaf deformation |  | +  RJ317** | +  RJ327** | + | +  RJ337** | +  RJ347** | + | + |
| E54944 | 01/07/2016 | Grunewald | leaf deformation, mottle, chlorotic ringspots and line pattern | *Aceria macrorhyncha, Eriophyes psilomerus* | +  RJ318** | +  RJ328** | + | +  RJ338** | +  RJ348** | + | + |
| E54945 | 01/07/2016 | Grunewald | mottle, chlorotic ringspots, line pattern |  | +  RJ319** | +  RJ329** | + | +  RJ339** | +  RJ349** | + | + |
| E54946 | 01/07/2016 | Grunewald | mottle, vein chloroses | *Eriophyes psilomerus* | +  RJ320** | +  RJ330** | + | +  RJ340** | +  RJ350** | - | + |
| E54948 | 01/07/2016 | Grunewald | mottle, chlorotic ringspots, line pattern |  | +  RJ321** | +  RJ331** | + | +  RJ341** | +  RJ351** | + | + |
| E54949 | 01/07/2016 | Grunewald | - |  | nt | - | - | - | - | - | - |
| E54950 | 01/07/2016 | Grunewald | - |  | nt | - | - | - | - | - | - |
| E54951 | 01/07/2016 | Grunewald | - |  | nt | - | - | - | - | - | - |
| E54952 | 01/07/2016 | Grunewald | - |  | nt | - | - | - | - | - | - |
| E54953 | 01/07/2016 | Grunewald | - |  | nt | - | - | - | - | - | - |
| E57720 | 13/05/2019 | Lehrgarten | chlorotic line pattern, mosaic |  | nt | +* | nt | + | + | nt | nt |
| E57721 | 13/05/2019 | Lehrgarten | mosaic, mottle |  | nt | +* | nt | + | + | nt | nt |
| E57722 | 13/05/2019 | Lehrgarten | chlorotic ringspots, mosaic |  | nt | +* | nt | + | + | nt | nt |
| E58060 | 30/06/2019 | Grunewald | mosaic |  | nt | +* | nt | + | + | nt | nt |

* RNA1 detected with a different primer pair [generic emaravirus primer pair acc. to Elbeaino et al. (2013), not mentioned in manuscript].

** RT-PCR products cloned and sequences for the genetic variability analysis.

nt = not tested
