# Supplementary material for: Next-Generation Sequencing Reveals a Novel Emaravirus in Diseased Maple Trees From a German Urban Forest
Source: Front Microbiol. 2021 Jan 8;11:621179. doi: 10.3389/fmicb.2020.621179 (PMC7819872; doi:10.3389/fmicb.2020.621179)
Supplement: Supplementary Table 2 — Estimates of evolutionary divergence among RT-PCR products from 10 maple trees and the original Acer+ (2014). (A) RT-PCR products amplified with primer-pair RNA1aF/R. (B) RT-PCR products amplified with primer-pair RNAbF/R. [file Table_2.DOC]

**Supplementary Table S2. Estimates of evolutionary divergence among RT-PCR products from 10 maple trees and the original Acer+ (2014).**

A. RT-PCR products amplified with primer-pair RNA1aF/R. B. RT-PCR products amplified with primer-pair RNAbF/R.

| A | RNA1a_E54938 | RNA1a_E54946 | RNA1a_E54937 | RNA1a_E54945 | RNA1a_E54943 | RNA1a_E54939 | RNA1a_E54934 | RNA1a_E54944 | RNA1a_E54948 |
| --- | --- | --- | --- | --- | --- | --- | --- | --- | --- |
| RNA1a_E54946 | 0.02 |  |  |  |  |  |  |  |  |
| RNA1a_E54937 | 0.016 | 0.01 |  |  |  |  |  |  |  |
| RNA1a_E54945 | 0.02 | 0.006 | 0.01 |  |  |  |  |  |  |
| RNA1a_E54943 | 0.02 | 0.006 | 0.01 | 0 |  |  |  |  |  |
| RNA1a_E54939 | 0.013 | 0.006 | 0.003 | 0.006 | 0.006 |  |  |  |  |
| RNA1a_E54934 | 0.013 | 0.006 | 0.003 | 0.006 | 0.006 | 0 |  |  |  |
| RNA1a_E54944 | 0.02 | 0.007 | 0.01 | 0.006 | 0.006 | 0.007 | 0.007 |  |  |
| RNA1a_E54948 | 0.02 | 0.006 | 0.01 | 0.006 | 0.006 | 0.006 | 0.006 | 0.006 |  |
| RNA1a_Acer+(2014) | 0.016 | 0.01 | 0.006 | 0.003 | 0.003 | 0.003 | 0.003 | 0.01 | 0.01 |

| B | RNA1b_Acer+(2014) | RNA1b_E54938 | RNA1b_E54944 | RNA1b_E54943 | RNA1b_E54948 | RNA1b_E54946 | RNA1b_E54935 | RNA1b_E54937 | RNA1b_E54939 | RNA1b_E54934 |
| --- | --- | --- | --- | --- | --- | --- | --- | --- | --- | --- |
| RNA1b_E54938 | 0.011 |  |  |  |  |  |  |  |  |  |
| RNA1b_E54944 | 0.007 | 0.011 |  |  |  |  |  |  |  |  |
| RNA1b_E54943 | 0.004 | 0.007 | 0.004 |  |  |  |  |  |  |  |
| RNA1b_E54948 | 0.004 | 0.007 | 0.004 | 0 |  |  |  |  |  |  |
| RNA1b_E54946 | 0.004 | 0.007 | 0.004 | 0 | 0 |  |  |  |  |  |
| RNA1b_E54935 | 0.004 | 0.007 | 0.004 | 0 | 0 | 0 |  |  |  |  |
| RNA1b_E54937 | 0.004 | 0.007 | 0.004 | 0 | 0 | 0 | 0 |  |  |  |
| RNA1b_E54939 | 0.007 | 0.004 | 0.007 | 0.004 | 0.004 | 0.004 | 0.004 | 0.004 |  |  |
| RNA1b_E54934 | 0.007 | 0.004 | 0.007 | 0.004 | 0.004 | 0.004 | 0.004 | 0.004 | 0 |  |
| RNA1b_E54945 | 0.007 | 0.004 | 0.007 | 0.004 | 0.004 | 0.004 | 0.004 | 0.004 | 0 | 0 |
